# Partial Submandibular Gland Resection in Deep Neck Surgery: An Assessment of Safety Using the LigaSure Exact Dissector

**DOI:** 10.1093/asjof/ojag041

**Published:** 2026-03-02

**Authors:** Paul N Afrooz, Natalie B Carter

## Abstract

**Background:**

Optimal cervicomandibular contour often necessitates management of subplatysmal structures, including the submandibular glands (SMGs). Partial SMG excision may enhance neck definition but remains controversial because of concerns regarding bleeding and other potential complications. The LigaSure Exact Dissector (Medtronic, Minneapolis, MN) is a bipolar vessel-sealing device that has been adopted by some surgeons for SMG management during aesthetic neck surgery.

**Objectives:**

To evaluate the short-term safety and postoperative complication profile of partial SMG excision using the LigaSure Exact Dissector during aesthetic neck lift surgery.

**Methods:**

A retrospective observational case series was conducted of patients undergoing partial SMG excision as part of primary neck lift procedures between August 2023 and January 2025. All SMG excisions were performed using the LigaSure Exact Dissector. Patient demographics and postoperative complications were recorded. Outcomes were analyzed descriptively, with a minimum follow-up of 3 months (mean follow-up, 5 months).

**Results:**

Of 123 neck lift procedures, 80 patients (65%) underwent bilateral partial SMG excision. Mean age was 58.7 years (range, 34-73 years); 57 patients (71%) were female and 23 (29%) male, with a mean body mass index of 24.5. Complications included seroma in 3 patients (3.75%), minor hematoma in 1 patient (1.25%), and transient lower-lip weakness in 4 patients (5%), all resolving within 8 weeks. No permanent nerve injury, sialoma, infection, or xerostomia occurred.

**Conclusions:**

Partial SMG excision using the LigaSure Exact Dissector was associated with a low rate of short-term complications. These findings suggest the technique can be safely performed in appropriately selected patients undergoing aesthetic neck lift surgery. Further comparative studies are warranted.

**Level of Evidence:**

4 (Therapeutic) 
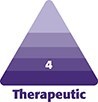

Neck lifting is a fundamental part of cervicofacial rejuvenation and essential to restoring youthful cervicofacial contours. Optimizing the cervicomandibular angle often requires comprehensive management of all anatomical layers of the neck, including subplatysmal fat, the anterior belly of the digastric muscles, and the submandibular glands (SMGs). Among these, the SMGs play a particularly significant role in defining neck contour.

The SMGs are paired salivary glands that lie deep to the platysma muscle within the submandibular triangle, bordered by the anterior and posterior bellies of the digastric muscle and the inferior margin of the mandible. Although it is a single lobe, the SMG can be anatomically divided by the posterior edge of the mylohyoid muscle into 2 lobes: the superficial and deep lobes. It is the superficial lobe of the gland that can become ptotic and enlarged, creating fullness in the submental region.

The SMG receives its principal blood supply from the facial artery and its cervical submental branch, with additional minor inputs from the lingual (deep lingual and sublingual) and occasionally direct external carotid branches.^1,2^ In addition to the rich vascular network, 3 nerves course near the SMGs: the marginal mandibular branch of the facial nerve, the hypoglossal nerve, and the lingual nerve. The marginal mandibular branch of the facial nerve is perhaps the most notable of the 3, as it is particularly at risk because of its close course and variability in location. It typically courses 1 to 2 cm below the mandibular border in a superficial plane, deep to the platysma but superficial to the facial vein.^3^ However, cadaveric studies have shown its course to be variable, with reports of it running above the inferior border of the mandible in 74% of specimens and below the inferior border of the mandible, dividing into 2 branches at the crossing point with the facial artery in 22% of specimens.^4^

Partial excision of the superficial lobe during neck lifting can dramatically enhance the definition and contour of the cervicomandibular angle. Despite its impact on aesthetic outcomes, SMG excision remains controversial and is often met with fear, largely because of its intraoperative technical demands and associated risks. Reported complications from partial SMG excision in neck lifting procedures include sialoma, hematoma, transient or permanent nerve injury, and, in 1 rare case, airway obstruction from bleeding in the deep neck.^5,6^ Therefore, partial SMG excision is considered on a case-by-case basis, weighing potential benefits against operative risk.

The LigaSure Exact Dissector (Medtronic, Minneapolis, MN) is a bipolar vessel-sealing device engineered for precision in confined anatomical spaces (Figure 1). Its fine, curved jaws allow controlled dissection and targeted energy delivery, enabling safe navigation around critical structures such as the marginal mandibular nerve and facial vessels. The device seals vessels up to 7 mm in diameter while minimizing lateral thermal spread, which may reduce nerve injury and postoperative morbidity. Originally developed for head and neck procedures, its application in aesthetic neck surgery may offer improved hemostasis, enhanced operative efficiency, and reduced complications compared with traditional monopolar and bipolar electrocautery.

**Figure 1.**
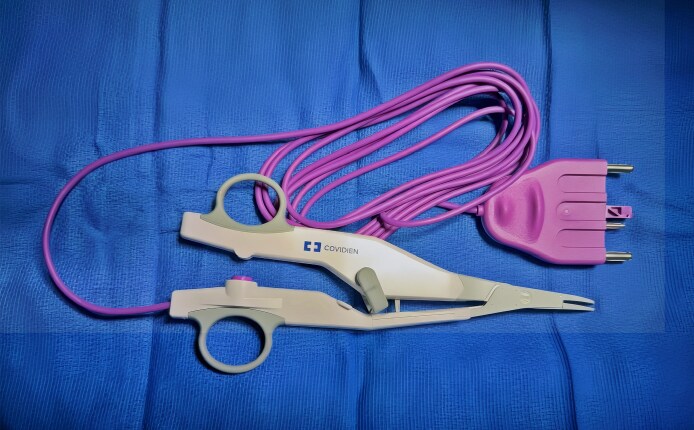
LigaSure Exact Dissector.

This article aims to demonstrate the safety and efficacy of partial SMG excision using the LigaSure Exact Dissector during neck lifting procedures.

## METHODS

### Study Design

This retrospective observational case series was conducted in accordance with the ethical standards of the institutional and/or national research committee and with the principles of the Declaration of Helsinki. IRB approval was not required for this study. Informed patient consent was obtained for inclusion in the study.

From August 2023 to January 2025, a total of 123 neck lift procedures were performed, largely in conjunction with full face and neck rejuvenation. Of these, 80 patients (65%) underwent bilateral partial SMG excision using the LigaSure Exact Dissector. Patients were included if they underwent primary neck lift surgery with bilateral partial SMG excision performed using the LigaSure Exact Dissector during the study period. Patients were excluded if partial SMG excision was not performed, if SMG management was performed using an alternative technique or energy device, if excision was unilateral, or if adequate postoperative follow-up data were unavailable for assessment of complications. To determine the need for SMG excision, all patients were evaluated by physical examination, with a final determination made intraoperatively, as described below.

### Statistical Analysis

Descriptive statistical analysis was performed to summarize patient demographics (Table 1), operative characteristics, and postoperative complications. Continuous variables are reported as means with ranges, and categorical variables are reported as frequencies and percentages. No inferential statistical analyses were performed, and no comparisons between groups were undertaken, because the study was designed as a descriptive retrospective case series. Statistical analysis was conducted using standard spreadsheet software.

**Table 1.** Patient Demographics (Entire Neck Lift Cohort, *n* = 123)

Characteristics	Value
Number of patients admitted for neck lift	123
Mean age, years (range)	59.5 (34-73)
Mean BMI (range)	26.4 (18.9-34.6)
Number of men (%)	29 (24)
Number of women (%)	94 (76)
Number of neck lift patients requiring partial SMG excision (%)	80 (65)

SMG, submandibular gland.

### Preoperative Assessment

**Figure 2.**
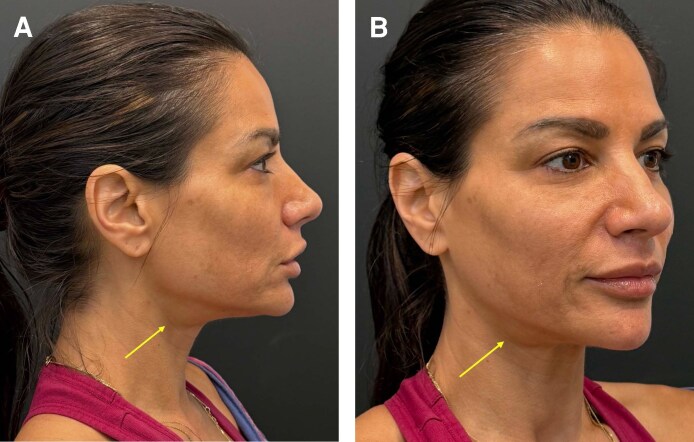
Preoperative photographs of a 59-year-old female demonstrating prominent submandibular gland contour. (A) Right profile view. (B) Right oblique view.

Large SMGs are frequently masked by subplatysmal fat and platysma muscle laxity. However, visual assessment of the neck can offer some initial insight into the underlying contributing factors of suboptimal cervicomental contour. Obliquity of the cervicomental angle and poor jawline definition in the oblique and profile views should raise the suspicion of excess volume of the subplatysmal contents. Therefore, physical examinations are essential. The SMGs are usually palpable as firm, discrete, mobile masses in the lateral submental triangle (Figure 2). SMGs that protrude inferior to the mandibular border on palpation are most likely contributing to suboptimal cervicomental contour.^7^ A final assessment can be done intraoperatively. During surgical exposure, the surgeon can directly evaluate gland position, bulk, and contribution to cervical contour. This step ensures that resection is performed only when the gland demonstrably contributes to submental fullness despite appropriate management of other structures (eg, fat compartments and digastric muscle fullness).

### Surgical Technique

A 3 to 3.5 cm incision is made in the submental crease or up to 1.5 cm posterior to the submental crease. Skin is separated from the underlying platysma with a small layer of preplatysmal fat left on the platysma muscle fascia. Maintaining this small layer of fat ensures a smooth postoperative contour while also maintaining the integrity of the platysma muscle fascia, which is essential for a strong suture repair. The midline is marked and incised with electrocautery from the submental incision to 3 to 4 cm below the hyoid. The platysma muscle is carefully dissected away from the subplatysmal fat using electrocautery. The dissection is carried laterally over the anterior belly of the digastric muscle. Subplatysmal fat is removed with electrocautery in accordance with the intended aesthetic objective. Following removal of subplatysmal fat, the SMG capsule is identified lateral to the anterior belly of the digastric muscles. The capsule is carefully incised on its medial aspect. The gland is dissected from the overlying capsule with a combination of electrocautery and blunt dissection. Adequate mobilization of the gland is essential for safe and precise resection. Once adequate mobilization has been achieved, the gland is grasped and pulled gently inferiorly and medially. The LigaSure Exact device is then used for incremental excision (Video). The SMG capsule was not repaired following partial excision in any case. Following SMG excision, the anterior belly of the digastric muscles is evaluated and contoured in accordance with the intended aesthetic objective. The subplatysmal space is irrigated copiously, and meticulous hemostasis is achieved. The medial borders of the platysma muscle are trimmed minimally to create a clean muscle and fascial edge. A midline platysmaplasty is performed with buried interrupted 3-0 polydioxanone sutures from superior to inferior.

Two 10 French round channeled Jackson–Pratt closed suction drains are placed. One drain is placed in the subcutaneous plane, and the other is placed in a subplatysmal plane. Drains are removed following output of <15 mL per 24 h period. No neurotoxin was injected into the gland stump.

## RESULTS

From August 2023 to January 2025, 123 neck lifts were performed largely in conjunction with full face and neck rejuvenation. Of 123 neck lifts performed, 80 patients (65% of total neck lifts) underwent partial SMG excision using the LigaSure device. Of the 80 patients, 57 (71%) were female and 23 (29%) were male. Among patients undergoing partial SMG excision, the average patient age was 58.7 (average age of females = 57.9, average age for males = 60.5), and the average BMI was 24.5.

The results were analyzed for 80 patients (mean age, 58.7 years; range, 34-73 years) who underwent bilateral partial SMG excision as part of a primary neck lift from August 2023 to January 2025. The average follow-up was 5 months (range, 3-16 months). Seromas occurred in 3 patients (4%), all within 7 days following drain removal. One seroma patient required placement of a percutaneous drain for 4 days. A minor subcutaneous hematoma occurred in 1 patient (1%), which was drained in the postanesthesia care unit by suture release of the postauricular skin and expression of the hematoma. Transient lower-lip weakness attributed to neurapraxia of the cervical branch occurred in 4 patients (5%), 3 of whom resolved within 4 weeks, and 1 of whom resolved at 8 weeks. No cases of sialoma, permanent nerve injury, infection, or xerostomia occurred (Table 2).

**Table 2.** Complications

Complications	*n* (%)
Transient lower-lip weakness	4 (5)
Seroma	3 (3.75)
Hematoma	1 (1.25)
Sialoma	0
Permanent nerve injury	0
Infection	0
Xerostomia	0

## DISCUSSION

SMG excision has remained a subject of controversy in aesthetic neck surgery since Connell first introduced subplatysmal dissection in 1987.^8,9^ Although its ability to enhance cervicomandibular definition is well recognized, concerns about technical difficulty, the vascularity of the gland, and the proximity of critical neurovascular structures have limited its widespread adoption. Many surgeons historically avoided SMG manipulation because of the perceived increase in operative time and the risks of bleeding or nerve injury.

Baker described subplatysmal surgery as “aggressive” and “radical,” arguing that potential risks outweighed aesthetic benefits.^10^ Similarly, Mendelson and Tutino reported a case of catastrophic airway obstruction from deep neck bleeding following SMG excision, which required tracheostomy.^6^ These concerns underscored the need for vigilance and careful implementation. Subsequently, however, experienced surgeons demonstrated that SMG excision could be performed safely and effectively with meticulous technique using monopolar and bipolar cautery.^7,11^

The introduction of advanced vessel-sealing technology has further expanded the safety profile of SMG excision. The LigaSure Exact Dissector, introduced in 2018, is particularly well-suited to subplatysmal planes. With a 2 mm wide tip, 40° jaw curvature, and a sealing length of 20.6 mm, it provides excellent visibility, efficient vessel sealing in 2 to 4 s, and rapid tip cooling to minimize thermal spread. By permanently sealing vessels, lymphatics, and soft tissue bundles through collagen and elastin denaturation, the device improves intraoperative control.^12,13^ Its benefits have been validated in other head and neck and oncologic surgeries, where it has been associated with shorter operative times, reduced drainage volumes, and lower seroma rates.^14,15^

In aesthetic neck lifting, Basaran and Comer recently evaluated the LigaSure Atlas (Medtronic, Minneapolis, MN) device for partial SMG excision. They reported an average excision time of 3.8 min per gland with no cases of intraglandular bleeding or postoperative hematoma. Minor bleeding from surrounding vessels was easily managed, and only 3 cases of seroma were observed. Temporary lower-lip weakness occurred in 7 patients, with all cases resolved within 6 months. These results support the device as a safe and efficient adjunct for SMG resection.^16^

Our own experience mirrors that of Basaran and Comert and Buckner and Shah, who reported excellent hemostasis using the Atlas handpiece and Sonicision Dissector (Ethicon, Cincinnati, OH), respectively.^16,17^ Unlike their series, our experience utilizes the Exact Dissector, which some may consider bulkier; however, we found its large, curved jaws advantageous for controlled access to the gland in confined subplatysmal spaces. Additionally, the larger jaw surface area allows the surgeon to grasp, seal, and cauterize broader portions of glandular tissue at once, enhancing hemostasis and efficiency while minimizing the need for repeated activations. Prior to adopting LigaSure, the senior author (P.N.A.) performed partial SMG excision with monopolar cautery. Despite meticulous efforts, inadvertent intraglandular vessel encounters often led to brisk intraoperative bleeding, increasing operative time and stress. Since transitioning to bipolar vessel sealing, these intraoperative challenges have been virtually eliminated, allowing for more controlled and bloodless resections. Although no direct comparator group was included in this report, qualitative observation may suggest that bipolar vessel sealing offers reduced bleeding and improved operative efficiency compared with prior monopolar cautery use. This observation warrants further controlled studies to definitively establish differences in safety or efficacy relative to other energy modalities.

Transient lower-lip weakness attributable to neurapraxia of the cervical branch occurred in a small subset of patients and resolved in all cases within 8 weeks. Cervical branch neurapraxia is a recognized complication of face and neck lift surgery and may occur regardless of the energy modality used for subplatysmal dissection. The etiology of neurapraxia in these cases cannot be definitively determined and is likely multifactorial. Potential mechanisms include traction injury during platysma elevation and lateral neck dissection, as well as localized thermal or compressive effects during subplatysmal work. Importantly, in our experience, the observed rate of transient neurapraxia was comparable to rates encountered prior to adoption of the LigaSure Exact Dissector. As such, these events cannot be attributed specifically to use of the device. Although bipolar vessel-sealing technology is designed to limit lateral thermal spread relative to monopolar electrocautery, this study was not designed to evaluate comparative nerve injury risk across energy modalities. Further prospective and comparative studies would be required to better elucidate the mechanisms underlying transient nerve dysfunction during aesthetic neck surgery.

**Figure 3.**
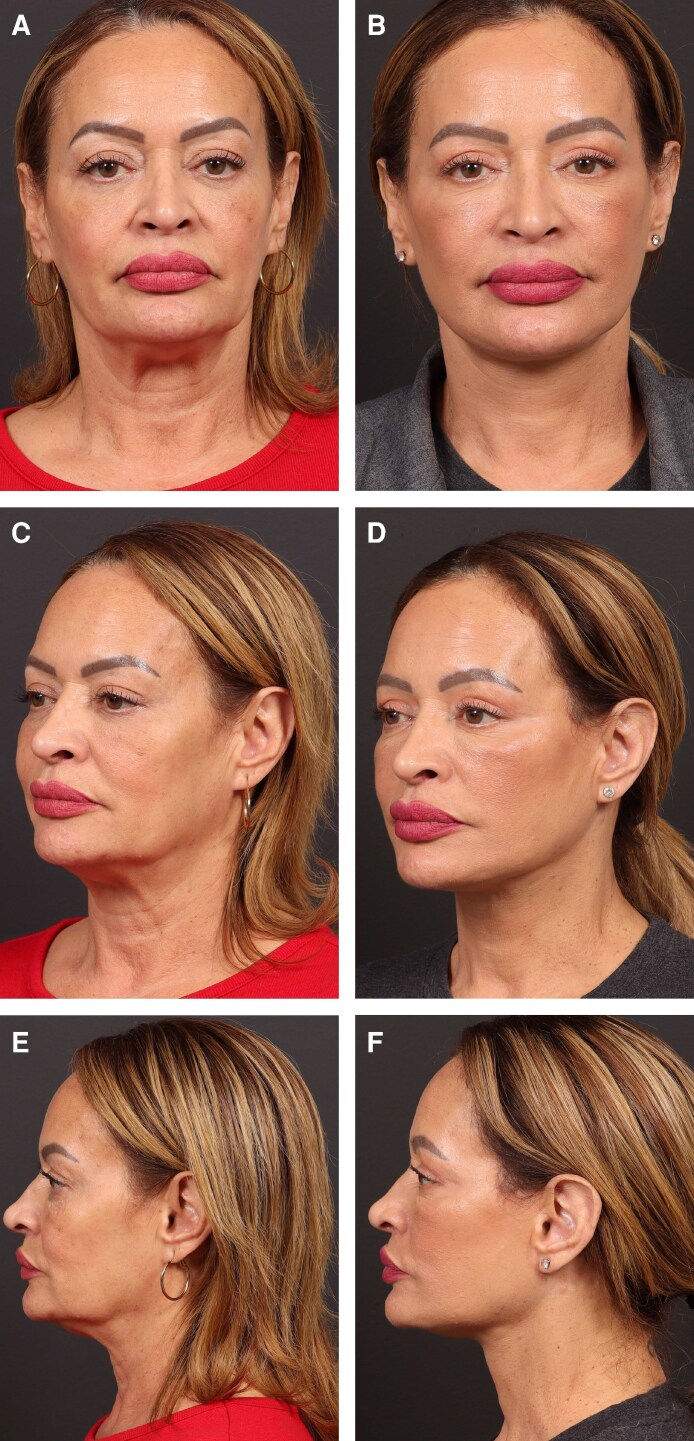
Preoperative and postoperative photographs of a 60-year-old female undergoing upper blepharoplasty, deep plane facelift, and deep neck lift with partial submandibular gland excision. (A) Preoperative frontal view. (B) Postoperative frontal view at 12 months. (C) Preoperative left oblique view. (D) Postoperative left oblique view at 12 months. (E) Preoperative left profile view. (F) Postoperative left profile view at 12 months.

SMG resection undoubtedly carries an increased risk for complications compared with more limited subplatysmal maneuvers. However, with careful patient selection, meticulous technique, and the adjunctive use of advanced bipolar vessel-sealing technology, these risks can be significantly mitigated. The device facilitates precise and hemostatic excision, reduces operative stress, and minimizes postoperative morbidity, thereby allowing surgeons to safely achieve improved cervicomandibular definition in appropriate candidates (Figure 3).

Although accessory bipolar vessel-sealing devices may serve as a valuable adjunct to improve safety and intraoperative control in experienced hands, several potential drawbacks merit consideration. Cost remains a primary limitation, as the disposable per-case expense of bipolar vessel-sealing devices is higher than that of standard monopolar electrocautery. In addition, use of the LigaSure Exact Dissector requires a compatible proprietary energy generator, representing an additional upfront capital investment that may limit adoption in certain practice settings. Adoption may therefore depend on surgeon preference, institutional resources, and the perceived value of SMG management in aesthetic neck surgery. Although the initial investment for the device and per-case consumables may be higher, many surgeons that value SMG reduction may find the tradeoff worthwhile because of enhanced intraoperative control, decreased bleeding, and potentially shorter operative times. The ability to seal vessels and lymphatics quickly can improve efficiency and reduce surgeon stress in complex neck dissections. From an ergonomic standpoint, the LigaSure Exact Dissector is bulkier and less familiar than a standard monopolar cautery device, which may require a brief period of surgeon acclimatization. However, in our experience, use of the device did not necessitate an increase in incision length and did not meaningfully impair visualization within the subplatysmal plane. Although alternative energy devices may offer different handling characteristics, the larger jaw profile of the Exact Dissector did not present prohibitive technical challenges in confined spaces. Ultimately, device selection should be individualized, weighing cost, ergonomics, and surgeon familiarity against the potential benefits of bipolar vessel sealing. Comparative studies are needed to more definitively evaluate relative advantages and limitations among available energy modalities.

This study has several limitations. Its retrospective design and single-surgeon experience limit generalizability and introduce potential selection and performance bias. The cohort size is modest, and follow-up duration is variable, precluding robust assessment of long-term outcomes. Additionally, no validated objective or patient-reported aesthetic outcome measures were employed; therefore, conclusions are limited to short-term safety and complication rates. Importantly, this study lacks a control or comparator group. As such, it cannot be determined whether the observed complication profile is attributable specifically to the LigaSure Exact Dissector or whether similar outcomes might have been achieved using alternative bipolar energy devices or conventional electrocautery techniques. Future prospective, comparative, and multi-institutional studies incorporating standardized outcome measures are necessary to more definitively evaluate the relative safety and efficacy of LigaSure-assisted partial SMG excision.

## CONCLUSIONS

Partial SMG excision remains a technically demanding component of aesthetic neck surgery. In this retrospective series, the use of the LigaSure Exact Dissector for partial SMG excision in carefully selected patients undergoing neck lift was associated with a low rate of postoperative complications and no cases of permanent nerve injury, sialoma, infection, or xerostomia during the follow-up period. Transient lower-lip weakness and seroma formation were infrequent and resolved with conservative management. Although longer-term follow-up and comparative studies are needed, these findings suggest that LigaSure-assisted partial SMG excision can be performed safely as part of aesthetic neck lifting in appropriately selected patients.
